# Micro-RNAs With Prognostic Significance in Gallbladder Cancer: A Systematic Review and Meta-Analysis

**DOI:** 10.7759/cureus.55515

**Published:** 2024-03-04

**Authors:** Rahul Saxena, Sarath Krishnan M P, Christhunesa S Christudass, Anil Chauhan, Vivek S Malik, Amit Gupta, Sweety Gupta, Akhil Anthwal, Bela Goyal

**Affiliations:** 1 Biochemistry, All India Institute of Medical Sciences, Rishikesh, Rishikesh, IND; 2 Neurological Sciences, Christian Medical College Vellore, Vellore, IND; 3 Telemedicine, Postgraduate Institute of Medical Education and Research, Chandigarh, IND; 4 Telemedicine, Centre for Evidence Synthesis and Public Policy, All India Institute of Medical Sciences, Rishikesh, Rishikesh, IND; 5 General Surgery, All India Institute of Medical Sciences, Rishikesh, Rishikesh, IND; 6 Radiation Oncology, All India Institute of Medical Sciences, Rishikesh, Rishikesh, IND

**Keywords:** hazard ratio, overall survival (os), prognostic significance, micrornas (mirnas), gallbladder cancer (gbc)

## Abstract

Gallbladder cancer (GBC) stands out as one of the most widespread malignancies impacting the biliary tract globally. Despite increasing interest, to the best of our knowledge, no meta-analysis has been undertaken to amalgamate the existing data concerning the prognostic significance of micro-RNAs (miRNAs) in GBC in comparison to studies on miRNAs in other cancers. Hence, this systematic review and meta-analysis aimed at determining the prognostic significance of miRNAs in GBC patients. Comprehensive literature searches were conducted across PubMed, Cochrane Library, Ovid, Scopus, and Science Direct databases. Studies that evaluated the association between miRNAs and overall survival in GBC patients were included. Random-effect meta-analysis was employed to pool hazard ratios (HRs) and their 95% confidence intervals (CIs) across studies. A total of 15 studies, encompassing 16 miRs, were included for our analysis. The pooled analysis revealed that a high expression of miR-204, miR-7-2-3p, miR-29c-3p, miR-125b, miR-20a, miR-139-5p, miR-141, miR-92b-3p, miR-335, and miR-372 was significantly associated with poor prognosis and increased risk (HR>1 and the upper bound of the 95% CI>1). Additionally, these miRNAs were associated with the overall survival (HR = 1.56, 95% CI = 0.91-2.20, *I^2^* = 91.82%). Significant heterogeneity was observed and could be attributed to the limited number of studies available on the GBC and significant reliance on quantitative real-time PCR for the detection of miRNAs. In conclusion, specific miRNAs exhibit prognostic significance in GBC, with potential implications for patient stratification and targeted therapeutic interventions. However, due to the heterogeneity among studies, these findings should be interpreted cautiously and validated in larger cohorts.

## Introduction and background

Gallbladder cancer (GBC) presents a notable challenge within the sphere of gastrointestinal oncology, largely due to its pronounced lethality that positions it among the most fatal malignancies globally [1]. While the scientific community has made progress in understanding and treating GBC, its survival rates remain alarmingly low compared to other prevalent cancers. The underlying molecular complexity and predisposing risk factors further complicate its clinical management. It is often diagnosed at an advanced stage, leading to limited treatment options, and resistance to standard therapies makes it an especially difficult cancer to tackle [2]. Traditional prognostic tools and methodologies have often proven inadequate in offering a precise prediction of disease course, leaving clinicians grappling with limited therapeutic choices. The vast heterogeneity of GBC, characterized by diverse and intricate tumor origin, renders the task of predicting prognosis even more challenging [3]. Thus, there is an unequivocal clinical imperative for the identification of precise predictive models for GBC that can not only stratify patients according to prognosis but also serve as a compass for therapeutic decisions [4].

Micro-RNAs (miRs) are emerging as a promising and novel source of prognostic biomarkers in cancer research [5]. These small non-coding RNA molecules are approximately 22 nucleotides long, transcribed from DNA into RNA hairpins, and perform a post-transcriptional gene regulatory function through the binding of the 3'-UTR of target mRNAs [6]. This results in either the degradation of these target mRNAs or the inhibition of their translation [7]. With their involvement in a range of biological functions and their key roles in multiple known hallmarks of cancer, including initiation, development, and metastasis, miRs have been shown to hold significant prognostic value in a variety of human cancers, such as colorectal, breast, lung, and ovarian cancers [8]. The mechanistic role of miRs in GBC, albeit partially deciphered, offers enticing insights [9]. Some miRs exhibit tumor suppressor attributes [10], while others seem to adopt onco-promotive tendencies [11]. Harnessing these molecular insights could pave the way not only for enhanced prognostic tools but also for ground-breaking therapeutic strategies such as miR mimics or inhibitors, which bear the potential to redefine GBC treatment paradigms.

Recent advancements in the field of miR research have shifted the focus toward GBC, with an emphasis on understanding their role in prognosis and as potential therapeutic targets. Preliminary studies suggest that miRs could play a critical role in predicting the prognosis of patients with GBC [5]. Despite the growing interest in this area, to our knowledge, no meta-analysis has been conducted to consolidate the available data regarding the prognostic significance of miRs in GBC. Therefore, in this study, we carry out a systematic review and meta-analysis to enhance clinical translation, and comprehensively explore the prognostic implications of various miRs in patients with GBC. Through this investigation, we aim to establish a foundation for developing more accurate prognostic models for GBC and elucidate novel therapeutic strategies to combat this deadly disease.

## Review

This systematic review and meta-analysis was conducted according to the “Cochrane Handbook for Systematic Reviews of Interventions” [12] and Preferred Reporting Items for Systematic Reviews and Meta-Analysis (PRISMA) 2020 guidelines (PROSPERO registration number of the study is CRD42022373004) [13]. This study was based on published literature. Therefore, ethical approval from ethics committees was not required.

Search strategy

A meticulous literature search was executed across electronic databases including PubMed/Medline, Cochrane Library, Ovid, Scopus, and Science Direct on August 9, 2023. The search strategy employed Boolean operators: ("Gallbladder Carcinoma" OR "Gallbladder Cancer" OR "Gallbladder Neoplasms") AND ("miR" OR "microRNA" OR "miRNA"). In addition to this, we undertook a manual exploration of the reference lists within relevant articles to ensure comprehensive coverage.

Selection criteria

The selection of studies was grounded on specific inclusion criteria: (1) delineation of miRs relationship with prognostic importance in GBC, (2) specified the miR threshold and provided a clear explanation of the miR quantification, and (3) correlation of survival outcomes with singular miR expression. On the contrary, exclusionary criteria encompassed the following: (1) non-English publications, (2) literature such as case notes, correspondences, commentaries, conference proceedings, or review papers, (3) studies with a participant count below 30, (4) research predominantly on animal subjects or cancer cells, (5) investigation of miR genetic variations, (6) derived hazard ratios (HRs) from several miRs, and (7) research that did not provide adequate data to determine the HR and its 95% confidence interval (CI). Assessment of these criteria was conducted autonomously by three separate authors to ascertain meticulous selection. Any conflicting interpretations were reconciled through discussion. In cases of duplicate/revised publications, only the most recent and content-rich iteration was chosen for subsequent analysis.

Data extraction

Data from selected studies were independently extracted by two investigators using a predefined form. Any inconsistencies in the data collection were addressed and rectified by a third researcher. The extracted data encompassed the following: the first author's last name, year of publication, the miRs investigated, demographic origins of the subjects, research methodology, stage of tumor, number of cases, participant gender, follow-up duration in months, detection technique, sample type, threshold values, and outcome metrics. Specifically, the HR for miR expression related to overall survival (OS) and its associated 95% CI were extracted.

Quality assessment

The studies' quality was assessed using the Newcastle-Ottawa scale, following the guidelines set by the Cochrane Group for Non-randomized Studies Methods. We examined the risk of bias in three areas: selection, comparability, and outcome [14]. The studies were classified according to AHRQ guidelines (good, fair, and poor) based on the star count in each domain (including selection, comparability, and outcome) following established methods [14]. A study is deemed to be of "good" quality if it has 3 stars or more in the selection domain, at least 1 star in the comparability domain, and 2 stars or more in the outcome domain. A "fair" quality study has 2 stars in the selection domain, at least 1 star in the comparability domain, and 2 stars or more in the outcome domain. A study is considered "poor" quality if it has 0 or 1 star in the selection domain, 0 stars in the comparability domain, or 0 or 1 star in the outcome domain [14]. Only studies rated as "good" or "fair" in quality were incorporated into the subsequent analysis to ensure the reliability and robustness of this meta-analysis.

Statistical analysis

The association between miR expression and GBC prognosis was determined using the pooled HR along with its 95% CI. If the HR was greater than 1, it indicated a poor prognosis for those with increased miR expression. On the other hand, if the HR was less than 1, it suggested a positive prognosis for those with higher miR expression. The consistency of the pooled HR was assessed using both Cochran’s Q test and the Higgins *I*^2^ statistic to detect heterogeneity. A p-value below 0.05 was deemed statistically significant. When heterogeneity between studies was detected (with a p-value of less than 0.05 in Cochran’s Q test), a random-effects model was applied. If no such heterogeneity was evident, a fixed-effects model was utilized. All the statistical evaluations were carried out using StataCorp 2019 (Stata Statistical Software: Release 16, StataCorp LLC., College Station, TX), and any result with a p-value under 0.05 was taken as statistically significant.

Publication bias

“Publication bias” was evaluated using the funnel plot and Egger's regression test [12].

Results

**Figure 1:**
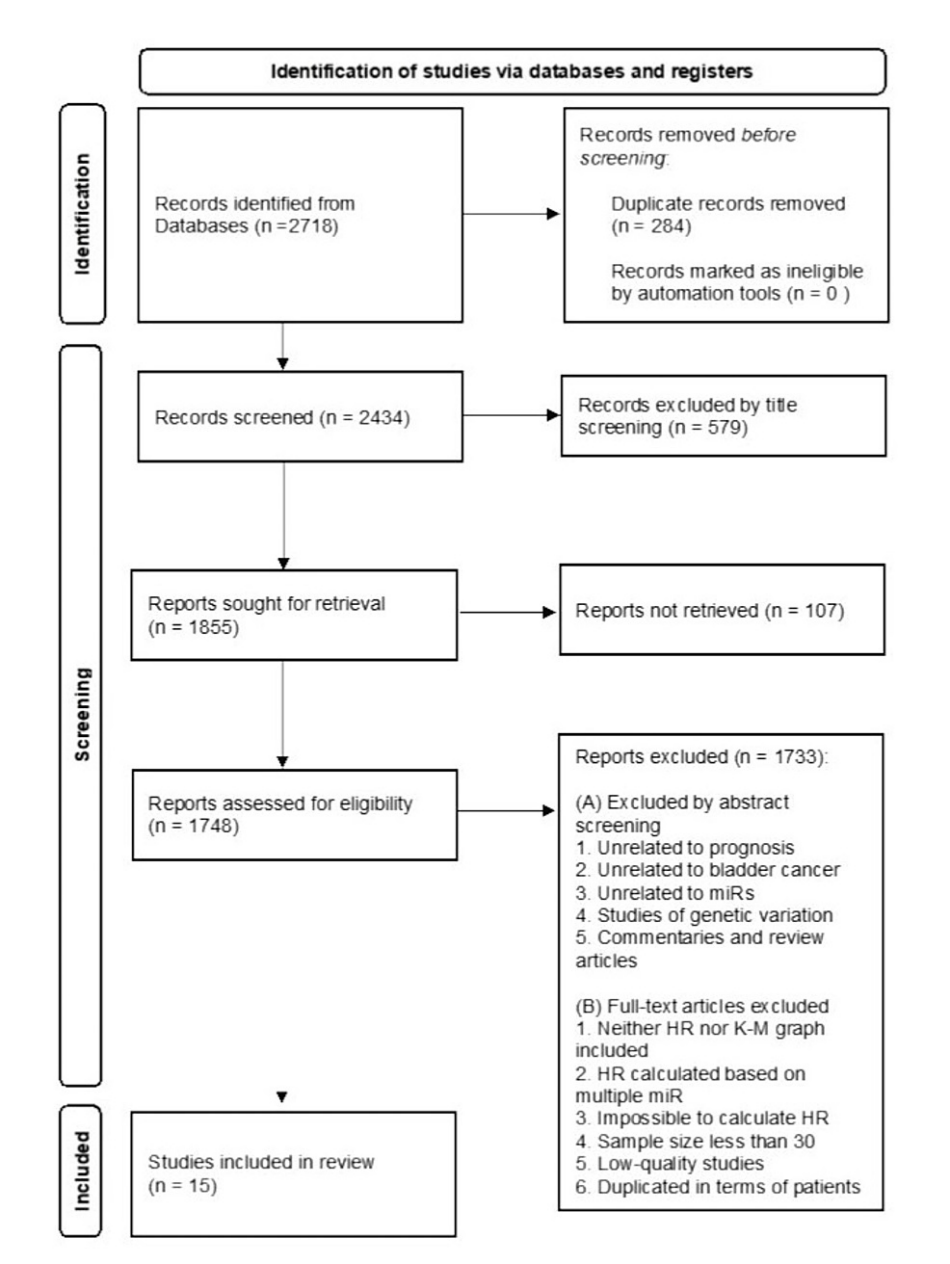
PRISMA flowchart illustrating the study selection process for the meta-analysis. HR, hazard ratio; K-M graph, Kaplan-Meier graph; miR, micro-RNA

From the initial literature search, 2,718 articles were obtained. Out of these, 284 were duplicates and were therefore removed. Upon reviewing the titles, 579 articles were discarded due to their nature as non-human studies, genetic variation studies, letters, case reports, reviews, commentaries, or other clearly unrelated studies. Additionally, 107 articles were excluded because their full text was not accessible. The articles that remained underwent further scrutiny through abstract and full-text reviews, leading to the exclusion of 1,733 articles. After a meticulous evaluation of the remaining potential articles, only 15 were deemed suitable for inclusion in this study and were subsequently used for data extraction (Figure 1). The 15 selected articles investigated the association between 16 specific miRs and GBC prognosis, meeting the requirements for inclusion in the meta-analysis. The progression of the study selection is visually represented in Figure 1.

Study characteristics

Between January 1, 2010, and July 1, 2023, all the selected studies were published. They delved into the prognostic implications of 16 distinct miRs for patients with GBC across different tumor stages. The primary method employed to determine miR expression was the quantitative real-time polymerase chain reaction. However, four of the studies utilized miRNA array analysis, and just one implemented next-generation sequencing (NGS) miRNA-seq. The majority of these studies analyzed miR expressions in tissue samples. Notably, one study examined miRs in both tissue and plasma, while another exclusively gauged miR levels in serum. A comprehensive breakdown of these 15 studies can be found in Table 1.

**Table 1:** Summary of the included studies in the meta-analysis detailing study characteristics, population, study design, TNM stage, sample size, gender distribution, follow-up duration, detection method, sample type, and Newcastle-Ottawa quality score. miR/miRNA, micro-RNA; NGS, next-generation sequencing; NR, not reported; qRT-PCR, quantitative real-time polymerase chain reaction; TNM, tumor-node-metastasis

Study	miR	Population	Study design	TNM stage	Case number	Gender (M/F)	Follow-up (month)	Detecting method	Detected sample	Quality score (Newcastle-Ottawa)
Zhang et al, 2021 [15]	miR-204	China	Case-control studies	I–IV	50	38/12	36	Non-coding RNA profiling by array, qRT-PCR	Tissue	Good
Lu et al [a], 2020 [16]	miR-7-2-3p	China	Case-control studies	I–IV	46	20/26	NR	miRNA array analysis, qRT-PCR	Tissue	Fair
Lu et al [b], 2020 [16]	miR-29c-3p				46	20/26	NR			
Yang et al., 2017 [17]	miR-125b	China	Case-control studies	I–IV	79	55/24	NR	qRT-PCR	Tissue	Good
Chang et al., 2013 [18]	miR-20a	China	Case-control studies	I–IV	67	27/40	NR	qRT-PCR	Tissue	Good
Chen et al., 2018 [19]	miR-139-5p	China	Case-control studies	I–IV	66	19/47	NR	qRT-PCR	Tissue	Good
Yang et al., 2022 [20]	miR-141	China	Case-control studies	I–IV	98	18/80	50	qRT-PCR	Tissue and plasma	Good
Lin et al., 2020 [21]	miR-92b-3p	China	Case-control studies	I–IV	94	35/59	NR	NGS miRNA-seq	Serum	Good
Zhang et al., 2015 [22]	miR-155	China	Case-control studies	I–IV	133	79/54	NR	qRT-PCR	Tissue	Good
Peng et al., 2013 [23]	miR-335	China	Case-control studies	I–IV	166	56/110	72	qRT-PCR	Tissue	Good
Zhou et al, 2017 [24]	miR-372	China	Case-control studies	I–IV	80	30/50	60	qRT-PCR	Tissue	Good
Shu et al., 2017 [25]	miR-29c-5p	China	Case-control studies	I–IV	40	14/26	36	miRNA array analysis	Tissue	Good
He et al., 2017 [26]	miR-143-5p	China	Case-control studies	I–IV	82	27/55	NR	qRT-PCR	Tissue	Good
Wang et al., 2017 [27]	miR-218-5p	China	Case-control studies	I–IV	82	27/55	NR	qRT-PCR	Tissue	Good
Bao et al., 2016 [28]	miR-101	China	Case-control studies	I–IV	53	18/35	NR	qRT-PCR	Tissue	Fair
Fatima et al., 2019 [29]	miR-335	India	Case-control studies	I–IV	50	8/42	NR	qRT-PCR	Tissue	Good

Micro-RNAs and prognosis

**Figure 2:**
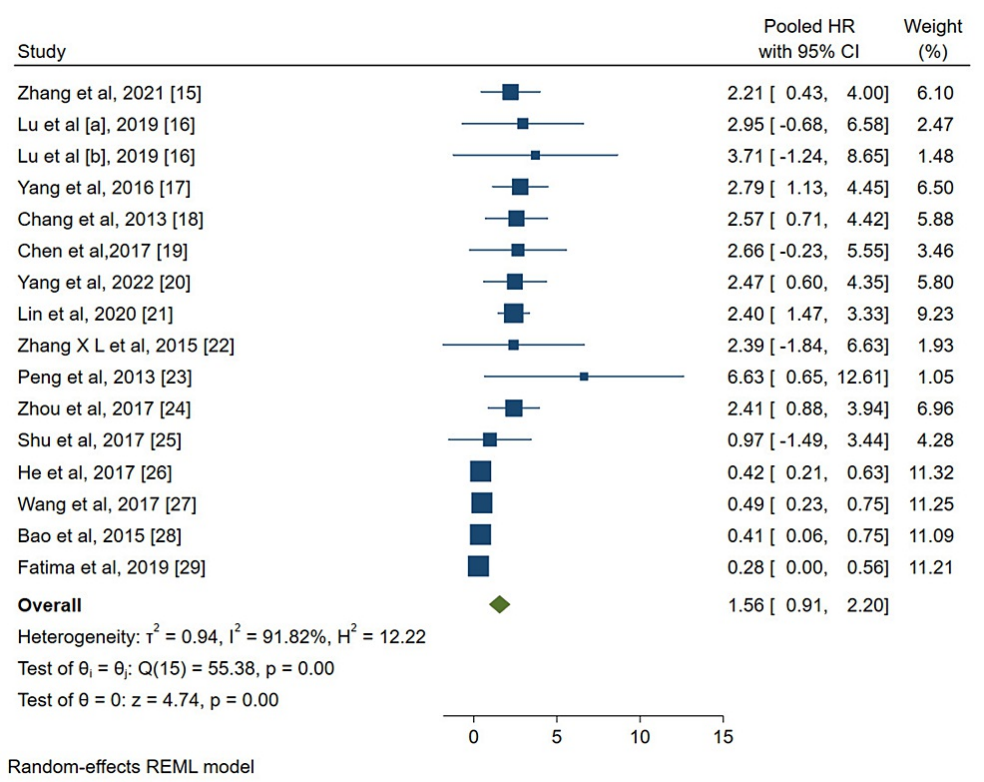
Visualization of the relationship between specific micro-RNA expression and prognosis in GBC patients based on data from 16 articles evaluating 17 distinct micro-RNAs as potential prognostic markers. CI, confidence interval; GBC, gallbladder cancer; HR, hazard ratio; REML, restricted maximum likelihood

The forest plot displayed results on an X-axis ranging from -5 to 15, with a reference line set at HR=1, to determine the relative risk of the included studies. High expressions of miR-204 [15], miR-7-2-3p, [16] miR-29c-3p [16], miR-125b [17], miR-20a [18], miR-139-5p [19], miR-141 [20], miR-92b-3p [21], miR-155 [22], miR-335 [23], and miR-372 [24] were associated with unfavorable prognosis (HR>1). Conversely, high expressions of miR-29c-5p [25], miR-143-5p [26], miR-218-5p [27], miR-101 [28], and miR-3355p [29] were associated with favorable prognosis (HR<1). Synthesizing the collective data, the pooled HR stood at 1.56 with a 95% CI ranging from 0.91 to 2.20. This suggests that while certain miRs might have a moderate association with prognosis, the overall effect across the studies is not strong, and there is a considerable overlap of CIs around the null value (HR=1). Heterogeneity testing, used to evaluate the consistency of results across different studies, revealed a substantial *I*^2^ value of 91.82%. This high percentage indicates significant variability among study results, which was further corroborated by *τ*^2^ = 0.94 and *H*^2^ = 12.22 values. Such disparities suggest that certain external factors, apart from random chance, might be influencing the observed outcomes. No same miRs were assessed by two or more studies (Figure 2).

Funnel plot for publication bias

**Figure 3:**
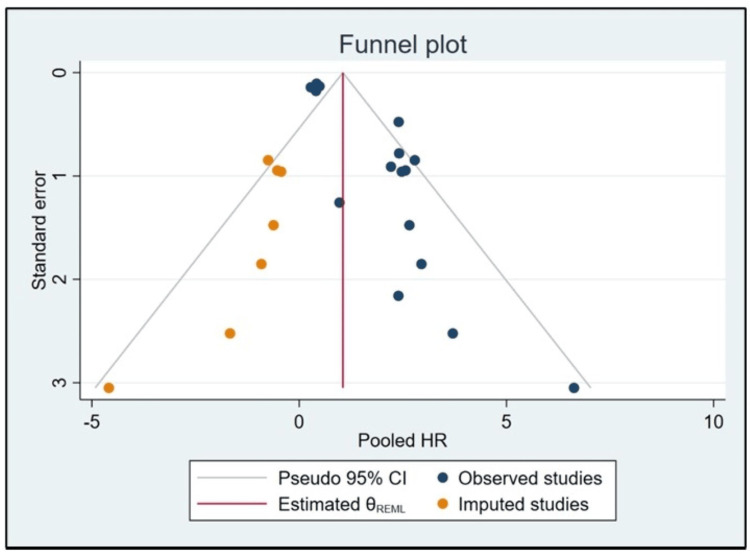
Funnel plot illustrating the distribution of 16 studies based on the pooled HR and standard error. The symmetry of the plot suggests an absence of notable publication bias, while the presence of outliers indicates potential study-specific heterogeneity. CI, confidence interval; HR, hazards ratio; REML, restricted maximum likelihood

To assess the potential existence of publication bias within the analyzed studies, a funnel plot was generated. For analysis purposes, we considered Lu et al. [a], 2020 [16] and Lu et al. [b], 2020 [16] as two separate studies, thus making a total of 16 studies. This visualization utilized the pooled HR on the X-axis, with values ranging from -5 to 10, and the standard error delineated on the Y-axis, ranging from 0 to 3. The funnel plot exhibited symmetry, indicating a lack of evident publication bias across the incorporated studies. The presence of outliers was constituted by the five observed studies and the imputed one. Such outliers may be indicative of inherent heterogeneity or unique attributes of these particular studies rather than a definitive manifestation of bias (Figure 3).

Galbraith plot

**Figure 4:**
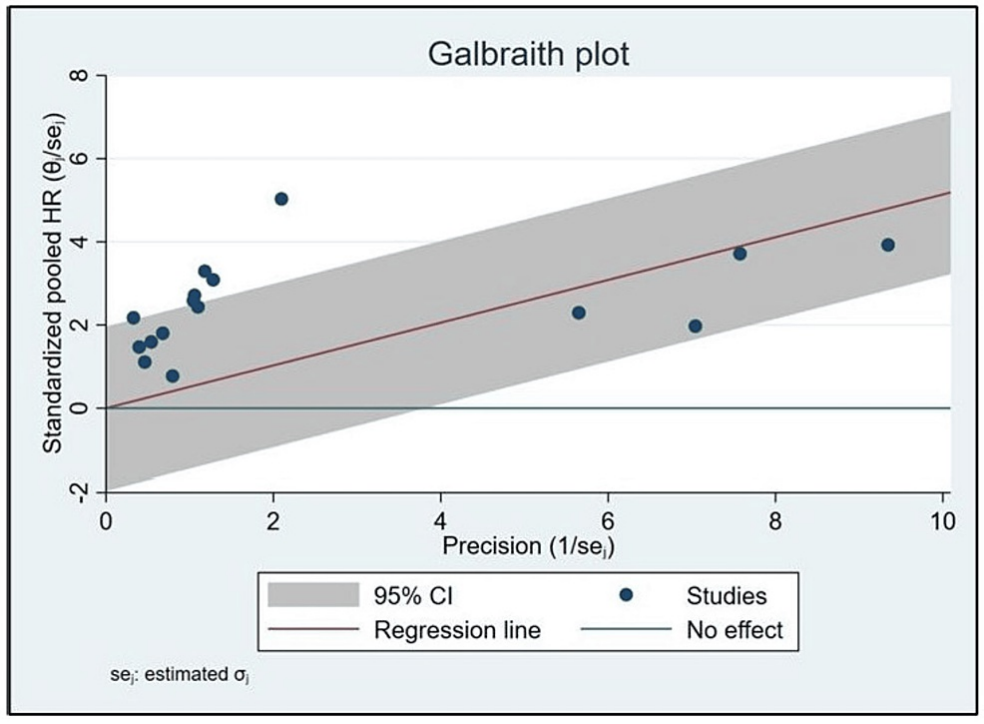
Galbraith plot displaying the relationship between standardized pooled HRs and precision for 16 selected studies. The plot highlights significant heterogeneity, with multiple studies deviating from the expected regression line and the 95% CI, indicating potential variances beyond sampling errors. CI, confidence interval; HR, hazard ratio

Utilizing the Galbraith plot as a diagnostic tool to discern potential heterogeneity within the selected studies, distinct distributional characteristics were observed. For analysis purposes, we considered Lu et al. [a], 2020 [16] and Lu et al. [b], 2020 [16] as two separate studies, similar to funnel plot. The ordinate represented the standardized pooled HRs, delineated between -2 and 8, while the abscissa illustrated precision, ranging from 0 to 10. Notably, of the 16 scrutinized studies, four were discernibly positioned below the regression trajectory, suggestive of HRs that might be statistically lower than their associated precision would predict. In contrast, a preponderance (12 out of 16 studies) was found above this trajectory, insinuating a consistent reporting of HRs in excess of anticipatory values predicated on their precision. The observed dispersion connotes significant heterogeneity, further corroborated by the deviation of multiple studies beyond the 95% confidence bounds. Such visual discernments accentuate the imperative to probe into contributory factors engendering the observed variances, transcending mere sampling inconsistencies (Figure 4).

Discussion

Micro-RNAs have garnered increasing attention in the field of oncology for their pivotal roles in the post-transcriptional regulation of gene expression and their potential impact on various cellular processes, including cell proliferation, differentiation, and apoptosis [30]. As a result, aberrant expression of certain miRNAs has been linked to tumorigenesis and the progression of various malignancies, positioning them as promising prognostic markers [6,8]. Their stable presence in bodily fluids and ease of detection also make them attractive diagnostic markers. In GBC, a malignancy renowned for its elusive early detection and poor prognosis, there is a growing need for reliable biomarkers that can guide therapeutic strategies and offer insights into the disease’s trajectory. Recognizing the potential significance of miRNAs as prognostic markers for GBC, we undertook this systematic review and meta-analysis to provide a comprehensive evaluation of the current evidence, striving for insights that can enhance our understanding and guide clinical decision-making.

Our systematic review discerned a geographical predominance in observational studies on GBC, with a notable majority stemming from China and a singular significant study from India. This geographical concentration is not coincidental but rather echoes the epidemiological trends identified in the GLOBOCAN 2020 data, wherein both China and India emerged as regions with elevated GBC prevalence [31]. The heightened incidence in these regions can be attributed to a combination of factors. Dietary habits, specifically the consumption of preserved foods rich in nitrosamines, are known risk factors for GBC and are prevalent in these territories [32]. Furthermore, chronic infections such as Salmonella typhi, which have higher incidences in these countries, have been implicated in the development of gallstone disease - a predominant risk factor for GBC [33]. Additionally, genetic predispositions, coupled with environmental and lifestyle factors prevalent in these densely populated regions, further amplify the risk of GBC. The concentration of observational studies in these regions underscores the urgency and relevance of understanding the molecular underpinnings of GBC, particularly in the context of miRs, to address this significant public health concern.

In the methodologies employed across the included studies, our review identified a significant reliance on quantitative real-time PCR (qPCR) for the detection of miRs, with only one study adopting NGS as the detection method. The dominance of qPCR can be understood given its accessibility, rapidity, and cost-effectiveness. However, in qPCR analysis, the selective examination of specific miRNA transcripts, as opposed to a comprehensive miRNA profile, can lead to observed heterogeneity. NGS, in contrast, offers a broader and more comprehensive view of miRNA profiles and might detect nuanced variations that are often overlooked in qPCR. Therefore, to ensure a holistic understanding of the prognostic significance of miRs in GBC, it is imperative to advocate for broader adoption of NGS in future research.

Our analysis encountered a pronounced heterogeneity in the miRs evaluated across different studies. This high variability can be primarily attributed to the limited number of studies available on the subject. With scant research on the topic, there exists no standardized set of miRs to investigate, leading individual studies to explore varied, and often distinct, miR candidates. This lack of consensus results in a diverse array of miRs being examined, consequently leading to the observed heterogeneity. Such heterogeneity, while expected in the nascent stages of a research area, underscores the pressing need for larger, collaborative efforts that can pool resources and knowledge. Establishing a more uniform approach to miR selection will not only reduce such disparities but also enhance the reliability and validity of prognostic findings in GBC. Additionally, fostering a global research perspective by including studies from diverse geographical regions will provide a more balanced and comprehensive data set, thereby elevating the robustness and generalizability of our conclusions.

A pivotal finding in our meta-analysis is the observed odds ratio exceeding 1, reinforcing the potential utility of miRs as biomarkers in GBC prognosis. This observation lends credence to the hypothesis that miRs can indeed be instrumental in the risk stratification of GBC. However, the resultant HR from the gathered data is notably diminished, an outcome heavily influenced by the heterogeneity as discussed above. This reduced HR can be attributed to the limited number of studies available and, by extension, the smaller patient cohorts within these studies. One cannot overstate that the paucity of GBC patients in studies is not just a reflection of the disease's rarity but also the unfortunate reality that GBC is often diagnosed at advanced stages, creating a distinct challenge for research. Moreover, the inherently aggressive nature of GBC results in reduced OS rates, a characteristic intrinsic to the disease and not a limitation of our study. Thus, it is crucial to understand that an effective prognosis offers a roadmap, elucidating the likely progression of GBC in individual patients. This allows healthcare professionals to anticipate, with greater accuracy, how patients sharing the same disease characteristics may respond to specific therapeutic interventions. The ability to predict not only the course but also the therapeutic response holds invaluable implications for patient care, potentially facilitating more tailored and efficacious treatment regimens for GBC sufferers. Therefore, while the reduced HR highlights challenges in the current literature, it simultaneously accentuates the importance and urgency of advancing miR-based prognostic strategies for GBC.

In synthesizing the insights from our systematic review and meta-analysis, we staunchly advocate for the integration of NGS as the predominant tool for miR detection in GBC. The intricacies of miRs are closely intertwined with the genetic heterogeneity influenced by geographical factors. Studies have noted differential miR expression across disparate geographical regions, a phenomenon likely modulated by local environmental factors and genetic determinants [34]. Given this nuanced landscape, it is imperative that future research adopts a multi-centric approach, incorporating extensive sample sizes that encapsulate this geographic diversity. Drawing parallels with expansive drug trials, we envision comprehensive, nationwide biomarker trials specifically tailored to miR discovery in GBC. Only upon identification and validation of these miRs as potential biomarkers, should they be subjected to rigorous trials to assess their efficacy as prognostic markers. Our findings underscore the tremendous potential of miRs in the prognostic realm. However, before their potential can be fully realized in therapeutic interventions, a methodical, phased, and collaborative approach to research is mandated. Only with this depth of understanding can we truly harness the full potential of miRs in GBC treatment and management.

## Conclusions

In conclusion, miRs stand as a promising biomarker for the prognosis of GBC. Our systematic review and meta-analysis underscore the significant potential of miRs in shaping the trajectory of therapeutic strategies and insights into the progression of GBC. Despite observed heterogeneity in miR studies and a discernible geographical bias in research, the prominence of elevated odds ratios emphasizes the prognostic relevance of miRs in GBC. The application of NGS, coupled with a more inclusive and diverse research approach, is poised to provide deeper insights into the intricate realm of miRs. It is paramount that as a research community, we foster collaboration, embrace advanced methodologies, and advocate for larger, multicentric studies that can elucidate the nuanced role of miRs in GBC. As the frontier of GBC research expands, harnessing the diagnostic and therapeutic potential of miRs may well revolutionize the management and treatment of this challenging malignancy.
